# Detection of *Ampelovirus* and *Nepovirus* by Lab-on-a-Chip: A Promising Alternative to ELISA Test for Large Scale Health Screening of Grapevine

**DOI:** 10.3390/bios12030147

**Published:** 2022-02-27

**Authors:** Ilaria Buja, Erika Sabella, Anna Grazia Monteduro, Silvia Rizzato, Luigi De Bellis, Vito Elicio, Lilia Formica, Andrea Luvisi, Giuseppe Maruccio

**Affiliations:** 1 Omnics Research Group, Department of Mathematics and Physics, University of Salento, CNR-Institute of Nanotechnology, INFN Sezione di Lecce, Via per Monteroni, 73100 Lecce, Italy; ilaria.buja@unisalento.it (I.B.); annagrazia.monteduro@unisalento.it (A.G.M.); silvia.rizzato@unisalento.it (S.R.); giuseppe.maruccio@unisalento.it (G.M.); 2 Department of Biological and Environmental Sciences and Technologies, University of Salento, Via Monteroni, 73100 Lecce, Italy; erika.sabella@unisalento.it (E.S.); luigi.debellis@unisalento.it (L.D.B.); 3 Agritest s.r.l., Tecnopolis Casamassima, Km. 3, Strada Provinciale Ceglie Valenzano, 70010 Valenzano, Italy; v.elicio@agritest.it (V.E.); l.formica@agritest.it (L.F.)

**Keywords:** plant pathogens, biosensors, lab-on-a-chip, on-chip assays, electrochemical impedance spectroscopy

## Abstract

The *Ampelovirus* Grapevine leafroll-associated virus 3 (GLRaV-3) and the *Nepovirus* Grapevine fanleaf virus (GFLV) are pathogens reported in many grapevine-growing areas all over the world, main causal agents of grapevine leafroll disease and grapevine fanleaf disease, respectively. Prevention of virus spread thanks to rapid diagnosis of infected plants is a key factor for control of both diseases. Although serological (e.g., enzyme-linked immunosorbent assay-ELISA test) and molecular methods are available to reveal the presence of the viruses, they turn out to be quite expensive, time-consuming and laborious, especially for large-scale health screening. Here we report the optimization of a lab-on-a-chip (LOC) for GLRaV-3 and GFLV detection, based on an electrochemical transduction and a microfluidic multichamber design for measurements in quadruplicate and simultaneous detection of both targets. The LOC detect GLRaV-3 and GFLV at dilution factors more than 15 times higher than ELISA, providing a higher sensitivity in the detection of both viruses. Furthermore, the platform offers several advantages as easy-to-use, rapid-test, portability and low costs, favoring its potential application for large-scale monitoring programs. Compared to other grapevine virus biosensors, our sensing platform is the first one to provide a dose-dependent calibration curve combined with a microfluidic module for sample analysis and a portable electronics providing an operator-independent read-out scheme.

## 1. Introduction

**Figure 1:**
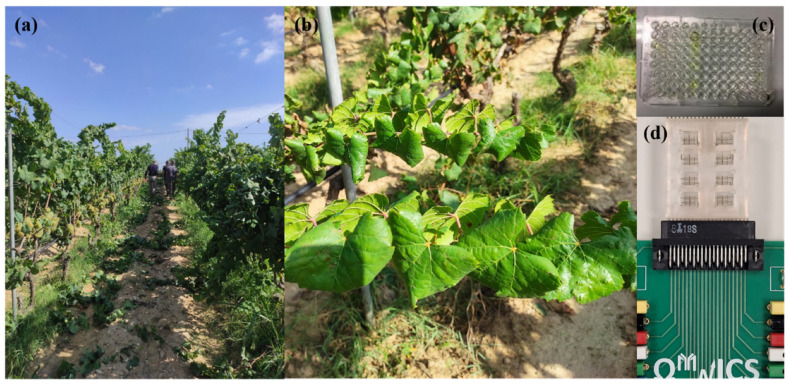
Grapevine leafroll disease and grapevine fanleaf disease diagnosis by ELISA and lab-on-a-chip (LOC) assays to detect Grapevine leafroll-associated virus 3 (GLRaV-3) and Grapevine fanleaf virus (GFLV). (**a**,**b**) Grapevines affected by GLRaV-3; (**c**) ELISA assay with diluted virus sources (1:3, 1:5, 1:10, 1:20, 1:50 and 1:100); (**d**) developed platform for portable, on-field LOC assays, with the sensor inserted in the PCB platform, ready to be connected to the potentiostat. Tests were carried on different dilutions (1:10, 1:20, 1:50, 1:100 for GLRaV-3 or 1:5, 1:10, 1:20, 1:50, 1:100 for GFLV).

*Ampelovirus* and *Nepovirus* genera include some of the most important viral pathogens of the grapevine, such as Grapevine leafroll-associated virus 3 (GLRaV-3) and Grapevine fanleaf virus (GFLV), respectively [1,2] (Figure 1). GLRaV-3 belongs to the family Closteroviridae, considered one of the most widespread pathogens associated with grapevine leafroll disease (GLD) [3], caused by various GLRaV-named viruses (from GLRaV-1 to –9), GLRaV-Pr, GLRaV-De and GLRaV-Car [4]. GLD affects red and white cultivars, with important economic repercussions [5,6]. GLRaV-3 symptoms are evident in red cultivars (which exhibit leaf reddening maintaining green venation) during late summer-fall, but not so apparent in white ones (with mild yellowing or chlorosis interveinal area) and become apparent on mature leaves during the post-véraison period. In both cases phloem disruption and downward rolling of leaf margins are present [3]. GFLV belongs to the Secoviridae family, and it is considered mainly responsible for grapevine fanleaf disease (GFD), which is one of the most severe virus diseases of grapevines [7]. *Nepoviruses* involved in fanleaf degeneration can cause symptoms such as leaf distortion, yellow mosaic close to primary veins, bright yellow vein banding on leaves, double nodes, and short and malformed internodes [8]. This depends mainly by the virulence of the virus isolate, the susceptibility of the grapevine variety, and environmental factors [9].

Transmission of GLRaV-3 and GLFV may occur via vegetative propagation materials, as well as vectors. GLRaV-3 can be transmitted through a semi-persistent way by mealybugs (Hemiptera: Pseudococcidae) and scale insects (Hemiptera: Coccidae) [10,11]. The nematode vector *Xiphinema index* is mainly responsible for GFLV transmission [9], allowing its worldwide distribution that can cause crop losses, affecting more than 80% of the crop.

Production losses due to GLRaV-3 and GFLV could have global scale repercussions, considering the lack of curative methods for plant viruses and the importance of wine production, which is estimated in more than 250 million of hectoliters [12].

Given the complexity of GLD and GFD control in the field, management strategies were used in different countries to address challenges associated with these diseases, requiring different goals such as: the education of growers, certification programs to provide disease-free planting material, the control of insect vectors and an extension of monitoring of GLD and GFD, over multiple years [3]. For European Union countries, specific rules [13,14,15] regulate the certification of marketable vines. These standards ensure the minimum level of quality of vine propagating material and their free circulation within the European Union.

Therefore, the use of preventive measures based of screening of plants (throughout diagnostic assay) is the main strategy to support marketing of healthy vines, demonstrating to be a valid tactic to counteract the spread of the virus in the vineyards [16]. Considering diagnostic approaches, ELISA and RT-PCR tests are complementary assays routinely used for the detection of GLRaV-3 and GFLV [16,17]. The first one is fast, reliable and adapted to test a large number of samples but it shows some limitations due to sensitivity and quality of antibodies. By contrast, although RT-PCR is useful due to its high sensitivity and robustness, it requires time, expertise and needs more economic investments [17].

A possible solution to improve effectiveness of low-cost diagnostic tests is given by the use of a Lab-on-a-Chip (LOC), as an innovative, smart and grower-friendly method for plant pathogen detection and monitoring [18,19,20,21,22]. For example, in a previous study, we reported a lab-on-a-chip method for rapid assay of *Xylella fastidiosa* subsp. *pauca* [20]. Concerning the detection of plant viruses, different strategies were adopted such as the DNA hybridization sensor based on screen-printed carbon electrodes modified with gold nanoparticles (AuNPs) for the selective detection of Citrus tristeza virus (CTV) [23]. Colloidal gold nanoparticles were used for antibody immobilization and the detection of Plum pox virus (PPV), in extracts from plum (*Prunus domestica*) and tobacco (*Nicotiana benthamiana*) leaves [24] through impedimetric immunosensors. Then the same authors reported an evolution of this platform, using glassy carbon electrodes as transducers for the detection of Prunus necrotic ringspot virus (PNRSV) [25]. Grapevine viruses were detected in [26,27,28,29] and are reviewed in more detail in the discussion section. Despite the global scale repercussions of grapevine pathogens, there is lack of portable tests for in situ analysis of these viruses. 

In this paper we report, for the first time, the detection of an *Ampelovirus* (GLRaV-3) and a *Nepovirus* (GFLV) through a LOC device connected to a portable potentiostat, with simultaneous electrochemical impedance measurements. Compared to the literature on grapevine virus biosensors, our sensing platform is the first one to provide a dose-dependent calibration curve combined with a microfluidic module for sample analysis and a portable electronics providing an operator-independent read-out scheme. Our LOC results are compared to a standardized serological method (ELISA) in which the same antibodies are used, suggesting that the proposed and portable technology could help in monitoring the spread of GLRaV-3 and GFLV, by providing a tool with features of low-cost, easy on-field use, and better performance than standard ELISA tests, despite being based on the same diagnostic principle. Indeed this is notably a versatile platform, which in previous versions was shown to be able to detect chemical and biochemical targets, spanning from clinical applications [30,31] to food safety control [32,33,34]. To our knowledge, our platform is the first example of portable impedance device for onsite detection of grapevine viruses. With this device it is also possible to acquire 16 measurements simultaneously, avoiding waste of time and reagents. The limited dimension of the platform and the possibility to easily integrate it into portable devices (laptops or smartphones) make our biochip suitable for health screening of grapevines.

## 2. Materials and Methods

### 2.1. Biological Samples 

Polyclonal antibodies against the viral coat protein (anti-GLRaV-3 and anti-GFLV IgG) and virus sources (lyophilized GLRaV-3- or GFLV-infected woody tissues collected from naturally infected grapevine plants) were obtained from commercial kits (Agritest, Valenzano, Bari, Italy). Negative controls (lyophilized GLRaV-3- and GFLV-free woody tissues collected from PCR-tested virus-free plants) were used as reference to tests. 

To evaluate the lot-specific detection limit of the ELISA commercial kit, dilutions of the virus source (1:3, 1:5, 1:10, 1:20, 1:50 and 1:100 for both viruses) were prepared with distilled water, then three replications of each dilution were screened. 

For LOC devices, tests were carried out with a dilution in Phosphate Buffered Saline (PBS) considering dilutions resulting negative for ELISA test (1:10, 1:20, 1:50, 1:100 for GLRaV-3 or 1:5, 1:10, 1:20, 1:50, 1:100 for GFLV, see Table 1). 

### 2.2. ELISA Assays

ELISA assays were employed as benchmark for the LOC performances. Specifically, DAS-ELISA was performed according to the manufacturer’s instructions (Agritest) using polyclonal antibodies against the viral coat protein, antisera dilutions and commercial buffers (Agritest). Values of the absorbance at 405 nm (OD_405_) were recorded 2 h after adding the substrate solution, using a PerkinElmer 2030 Multilabel reader Victor X5 (PerkinElmer, Turku, Finland). Normalized R values were defined as (OD-sample⁄OD-negative control) setting a threshold at R = 3.0 to discriminate between positive and negative samples (according to manufacturer instructions).

### 2.3. LOC Fabrication and Functionalization

**Figure 2:**
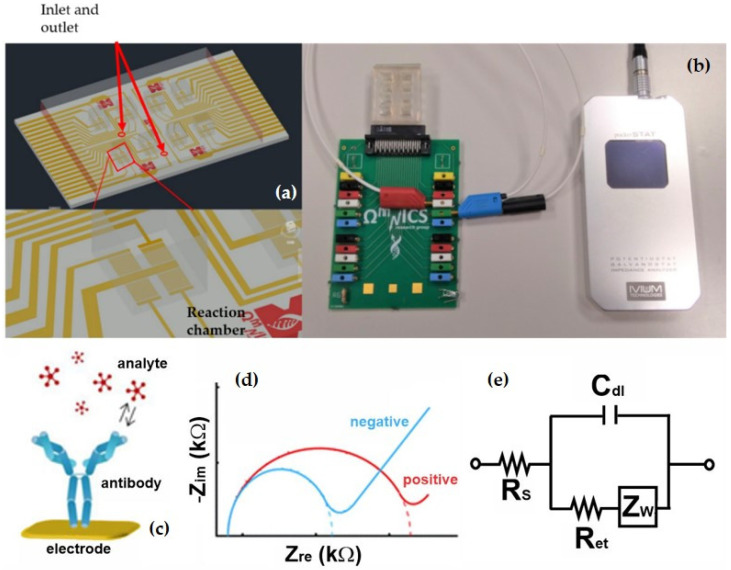
Schematic illustration of the LOC device optimized for the detection of GLRaV-3 and GFLV. (**a**) The device is coupled to a microfluidic module and inserted in (**b**) a PCB platform, communicating to IVIUM potentiostat for impedance analysis. Interdigited electrodes have 10 μm lines and spacing. (**c**) Analyte detection on the functionalized electrode surface and (**d**) resulting change in Nyquist curves with positive sample (red curve) compared to negative control (blue curve). (**e**) The recorded response can be modelled using a simplified Randles equivalent circuit comprising the resistance of the electrolyte solution R_s_, the Warburg impedance Z_w_, the double layer capacitance C_dl_ and the electron transfer resistance R_et_.

The LOC system used for GLRaV-3 and GFLV detection includes a PDMS (polydimethylsiloxane) microfluidic module with microchannels and 20 μL microchambers obtained by replica molding. The system has two sides (half chip), each one made of a central inlet and 4 peripheral outlet holes for the delivery of solutions. The layout of the interdigited microelectrodes array (Figure 2) allows simultaneous measurements in replicate (four per chamber) and on different samples (4 chambers per half chip). For enabling GLRaV-3 and GFLV detection in homogenized samples, interdigited electrodes were functionalized with highly specific antibodies (the same employed for ELISA tests). In more detail, the functionalization process starts with the overnight deposition of a mixed self-assembled monolayer (SAM) of mercaptoundecanoic acid (11-MUA) (Sigma-Aldrich, Burlington, MA, USA) and 2-mercaptoethanol (2-ME) (Sigma-Aldrich) in a ratio of 1:5 (0.2 mM of 11-MUA and 1 mM of 2-ME) followed by the activation of the COOH groups by incubation with N-hydroxysuccinimide (NHS) (Sigma-Aldrich) and N-ethyl-N-(3-di-methylaminopropyl) carbodiimide hydrochloride (EDC) (Sigma Aldrich) in ultra-pure water for 30 min, to form reactive N-hydroxysuccinimide esters. Successively, electrodes are incubated for two hours in a solution of Protein G (50mg/L) (Sigma-Aldrich) able to bind the antibodies, placed in contact with ethanolamine (1 M) (Sigma-Aldrich) for 20 min and passivated with Bovine Serum Albumin (Sigma-Aldrich) 1 mg/mL for 15 min, dissolved in PBS pH = 7 at room temperature, to saturate remaining free electrode sites. Then the chips are incubated with antibodies (Agritest), for an hour, diluted 1:1000 in PBS for GLRaV-3 and 1:500 for GFLV, with 0.03% sodium azide and finally, washed with PBS. All the functionalization steps are realized at room temperature.

### 2.4. LOC Analysis of Samples

Once the sensing devices have been produced, detection was achieved by impedance spectroscopy since target analyte binding on the functionalized electrode surface results in a measurable increase in the electron transfer resistance which can be correlated to its concentration [35,36,37]. More in detail, chambers were filled with a redox couple solution of hexacyanoferrate (II/III) K_3_[Fe(CN)_6_]/K_4_[Fe(CN)_6_] (1:1) at a concentration of 10 mM in order to perform electrochemical impedance measurements. A portable IVIUM Technologies (Eindhoven, The Netherlands) potentiostat (Figure 2) was employed to acquire impedance spectroscopy data by applying a sinusoidal 10 mV AC voltage in a range of frequencies from 10^5^ Hz to 0.1 Hz. The potentiostat was manually connected to different positions on the PCB in order to measure various electrodes and chambers recording 4 measurements per chamber in less than 12 min. To automate the procedure, a multiplexer can be integrated in the PCB adding the possibility to switch automatically over the transducer array. After optimization of the response of antibody-functionalized surfaces, electrodes were incubated with serial dilutions of GLRaV-3 and GFLV samples for 1 h, at room temperature, to allow the biorecognition, using four chambers per side (half chip) for measurements in quadruplicate, for each dilution, with a volume of 15 µL for each chamber. Subsequently, 1 mL of washing PBS solution was delivered into the system and chambers were filled with the redox couple solution to perform electrochemical impedance measurements. The same protocol was used for the incubation of negative control samples in order to test system’s ability to discriminate the presence and absence of the virus.

### 2.5. Statistical Analysis

All data were statistically analyzed and reported in the form of mean values with standard deviation. Comparisons between different experimental conditions were done using Student’s *t*-test. *p*-values < 10% (*), *p* < 1% (**), *p* < 0.1% (***) were considered statistically significant.

## 3. Results

### 3.1. ELISA Assay 

In ELISA tests, control samples had R = 1.00 whereas a threshold value R = 3.0 was used to discriminate positive samples. In this way, ELISA assays demonstrated that they were able to detect GLRaV-3 and GFLV up to dilution 1:5 and 1:3, respectively. No detections were achieved for higher dilutions (Table 1). 

### 3.2. LOC Assay

Electrochemical impedance biochips were employed to evaluate their ability to detect the viral infections and provide a portable analytical platform for on-field monitoring. LOC assays were performed with a dilution in Phosphate Buffered Saline (PBS) considering dilutions resulting negative in ELISA tests (namely 1:10, 1:20, 1:50, 1:100 for GLRaV-3 or 1:5, 1:10, 1:20, 1:50, 1:100 for GFLV). The resulting impedance spectra are shown below in the form of Nyquist plots where the real (Z_re_) and imaginary (-Z_im_) components of the complex impedance are plotted on the x and y axis, respectively. The reported impedance curves have been obtained by at least three repeated measurements and error bars represent the standard deviations. Information on electrode modifications and a quantification of sensor response to be correlated with the concentration can be obtained by modelling the system with a simplified Randles equivalent circuit (Figure 2e). The most relevant parameter for our analysis is the electron transfer resistance R_et_ estimated by the fit and approximately corresponding to the semicircle diameter. R_et_ increases progressively for consecutive molecular layers depositions on the electrode. Thus, R_et_ can be considered an indicative parameter for the functionalization and target detection phases and correlated to the analyte dilutions. 

**Figure 3:**
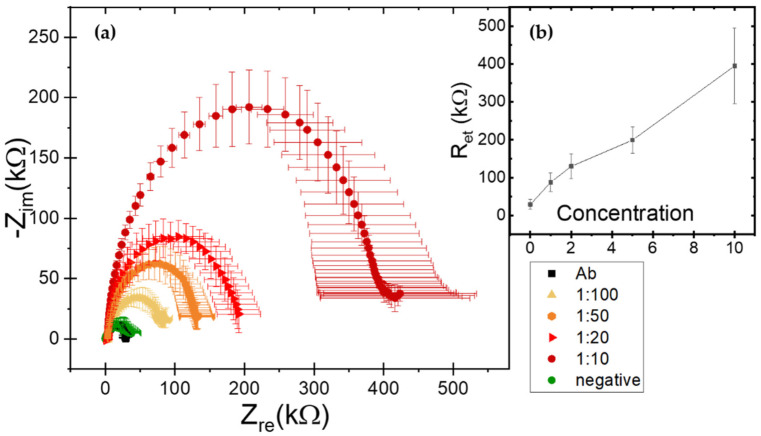
(**a**) Nyquist spectra in response to serial dilutions of GLRaV-3 virus source homogenate. Black curve is related to antibody functionalization. Negative sample response corresponds to green curve. Incubation with positive samples leads to higher impedance values, from 1:100 (yellow curve) to 1:10 (red curve) with an increased darkness in the curve color (**b**) Biosensor calibration curves for GLRaV-3 in terms of R_et_ (mean ± SD) vs. concentration expressed as 100 divided dilution factor. R_et_ was evaluated by means of fits with a simplified Randles equivalent circuit model.

**Figure 4:**
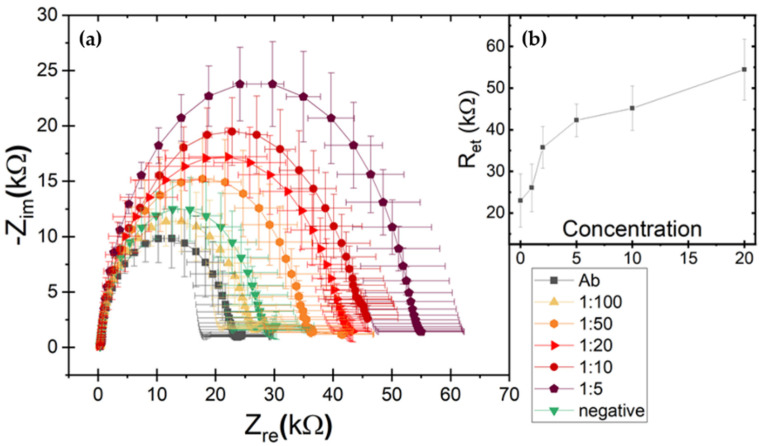
(**a**) Nyquist spectra in response to serial dilution of GFLVs virus source homogenate. Black curve is related to antibody functionalization. Negative sample response corresponds to green curve. Incubation with positive samples leads to higher impedance values, from 1:50 (orange curve) to 1:5 (purple curve) with an increased darkness in the curve color (**b**) Biosensor calibration curves for GFLV in terms of R_et_ (mean ± SD) vs. concentration expressed as 100 divided dilution factor. R_et_ was evaluated by means of fits with a simplified Randles equivalent circuit model.

As a first step, the baseline response of the biosensors after functionalization phase was quantified, in order to obtain the reference values associated with the antibodies anti-GLRaV-3 and anti-GFLV. Reproducible electron transfer resistance (R_et_) values around 30 ± 13 kΩ for GLRaV-3 and 23 ± 6 kΩ for GFLV were recorded (black curves in Figure 3a and Figure 4a, respectively). 

Successively, a calibration using serial dilutions of the GLRaV-3 virus source homogenate was performed. A remarkable decrease in impedance values was observed for higher GLRaV-3 dilution ratios, thus providing a robust demonstration of LOC diagnostic ability relying on biorecognition events at the interface between the functionalized electrodes and the solution. Indeed, the electron transfer resistance (R_et_) increased consequently to the absorption of molecular layers and analytes on the electrode surfaces and this process was concentration-dependent. The reason is that on bare electrodes redox reactions can easily take place, through the redox couple solutions, allowing electron transfer from the species in the solution to the electrodes and resulting in low impedance values. On the other hand, when analytes were detected and became attached on the electrode surface (target detection), the electron transfer process was hindered and this resulted in an increase in the R_et_ value. 

Quantitatively, in GLRaV-3 case, the incubation with 1:10 diluted samples resulted in a R_et_ value around 400 ± 100 kΩ (dark red curve in Figure 3a); a 1:20 dilution of the virus source sample resulted in R_et_ around 200 ± 34 kΩ (red curve); the 1:50 dilution gave R_et_ around 130 ± 33 kΩ (orange curve), while R_et_ was around 88 ± 25 kΩ for 1:100 samples (yellow curve) and 40 ± 24 kΩ in the case of the negative samples (green curve). Notably, the curve associated with 1:100 ratio still deviated significantly from antibody and healthy sample signals, demonstrating that impedance spectroscopy is very effective for monitoring biorecognition events on surface-modified electrodes and sensitive to different dilutions of GLRaV-3, discriminating between positive and negative samples up to a 1:100 dilution.

**Figure 5:**
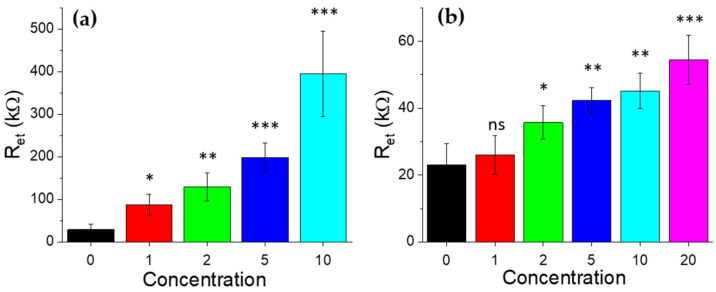
Statistical comparison among the LOC response at increasing (**a**) GLRaV-3 virus and (**b**) GFLVs virus concentrations (expressed as 100 divided dilution factor) with respect to the corresponding responses after antibody immobilization. *p*-values < 10% (*), *p* < 1% (**), *p* < 0.1% (***) were considered statistically significant. ns indicates no statistically significance.

Considering GFLV, a remarkable variation in impedance curves and R_et_ values was also observed. In particular, 1:5 dilution resulted in a R_et_ value around 54 ± 7 kΩ (purple curve in Figure 4a); 1:10 dilution gave R_et_ around 45 ± 5 kΩ (dark red curve); 1:20 dilution resulted in R_et_ ≈ 42 ± 4 kΩ (red curve); 1:50 dilution in R_et_ ≈ 36 ± 5 kΩ (orange curve) and 1:100 dilution in R_et_ ≈ 26 ± 6 kΩ (yellow curve). The negative sample curve exhibited a R_et_ ≈ 30 ± 6 kΩ (green curve). In this case, however, the curve associated with 1:100 diluted samples was still statistically compatible with curves corresponding to antibody immobilization and negative samples and could not be discriminated. Accordingly, to the observed uncertainty bars, the lowest detectable concentration was 1:50, as confirmed by the statistical analysis summarized in Figure 5. 

Concerning the dose-response calibration curves, for GLRaV-3 an almost linear trend was observed down to 1:100 dilution, while for GFLV till 1:50. Differences in detection limits among the two viruses could be ascribed to differences in the viruses’ dimensions and morphology. Indeed, GLRaV-3 particles are flexuous filaments, 1800 × 12 nm in size, helically constructed and contain approximately 10 protein subunits per turn of the helix, whit a pitch of about 3.5 nm [38]. GFLV are isometric particles with about 30 nm in diameter [9]. These differences could motivate the different signal values and statistical variations.

## 4. Discussion

Conventionally, laboratories around the world adopt traditional diagnostic techniques and, among these, ELISA assays are one of the most widespread for detection of grapevine viruses. Despite the extensive use of serological methods, these techniques have some limitations, since they are expensive, and require time and qualified personnel. For this reason, innovative tools suitable for field use are today required, especially if we consider the worldwide spread of phytopathological adversities, facilitated by the globalized market. To respond to this need, we developed a low-cost, portable LOC assay based on electrochemical impedance spectroscopy transducers and able to detect GLRaV-3 and GFLV at lower concentrations than ELISA tests. 

In literature, few (four) technological reports address the development of biosensors and lab-on-a-chip for detecting grapevine phytopatogens and in particular viruses, as summarized in Table 2. Two of them are based on changes in drain-source current [29] and whispering Gallery Mode Resonators [28] but do not provide a dose-response curve. Another recent study based on photoluminescence read-out instead reports a calibration curve, but this is strongly dependent on the operation point (read-out wavelength) since the relative signal values change significantly even for minor wavelength changes. Finally, [26] reported an assay with sensitivity of 100% vs. ELISA and 93% vs. PCR which is based on visual detection and thus can be more subject to be operator-dependent. In this respect, our sensing platform is the first one to provide a dose-dependent calibration curve combined with a microfluidic module for sample analysis and portable electronics providing an operator-independent read-out scheme. 

In Table 3, the results and performance of the two methods are summarized and compared, for both GLRaV-3 and GFLV detection. These findings place the LOC platform in a competitive position because of its lower limit of detection. Indeed, ELISA tests fail in detecting GLRaV-3 at dilution factors higher than 1:5, while the LOC platform is able to identify the pathogens down to 1:100 dilutions, showing a limit of detection 20 times better than ELISA. A similar improvement was observed for GFLV detection, with ELISA tests limited at 1:3 dilution factor and our LOC platform able to identify the pathogen down to 1:50 dilutions, corresponding to about 16 times improvement. The higher R_et_ values (and uncertainty bars) observed in GLRaV-3 can be attributed to higher inhomogeneity and the possible formation of some agglomerates in these samples (especially at higher concentrations) as compared to the GFLV case.

In addition to field-use possibility for the LOC platform, this increase in performance can have useful repercussions in terms of early detection, with particular reference to the support of self-diagnostic actions in the nursery sector. Furthermore, an interesting feature of the proposed system is the possibility to perform a multiplex and simultaneous antibody test, as an alternative to currently available multiplex PCR methods, detecting individual infections in a single assay. Especially considering nursery quality control, this method of analysis would allow evaluating and distinguishing the presence of certification and quarantine organisms simultaneously. Finally, as previously shown in Figure 1, LOC is simply inserted into a PCB platform that communicates results to a PC, thanks to the aid of a portable potentiostat. In terms of lifetime, the microchip can be functionalized with antibody and stored at 4 °C, up to 1 month with minor changes in the response. Regarding the costs, this platform is also competitive, especially considering that each device has a lab production cost of about EUR5, due mainly to the glass substrate and the metallization processes [20]. It is reasonable to think that the costs would be lower, producing 10,000 devices or more, making it economically competitive as well.

Considering the results achieved with this platform, LOC sensors are expected to be useful tools for nurseries/wine production companies and plant pathologists with the possibility to perform on-field analysis, save reagents and also expand its application for the detection of other grapevine viruses. 

## Figures and Tables

**Table 1:** ELISA results (+ = positive/detectable, − = negative/undetectable) on serial dilutions from 1:3 to 1:100 of virus sources for GLRaV-3 and GFLV. The R value was defined as R = OD_sample_/OD_negative control_.

Sample Dilution	GLRaV-3		GFLV	
	R	Result	R	Result
1:3	5.42	+	3.49	+
1:5	4.61	+	1.83	−
1:10	2.55	−	1.12	−
1:20	1.49	−	0.86	−
1:50	0.92	−	0.71	−
1:100	0.89	−	0.69	−
Negative control	1.00	−	1.00	−

**Table 2:** Comparison of operative conditions and performance among our biochip and the present literature on biosensors for grapevine pathogens detection.

Bibliographic Reference	Target	LOD	Sensing Element	Transduction	Fluidics
Our biochip	GLRaV-3- or GFLV-infected woody tissues	1:50 for GFLV; 1:100 for GLRaV-3	Ab immobilized on gold microelectrodes	Electrochemical impedance	Yes
Byzova et al., 2018	GLRaV-3-, GLRaV-1-, GVA- and GFLV-infected leaf tissues	Sensitivity of 100% vs. ELISA; sensitivity of 93% vs. PCR;	Immobilized antibody–antigen–GNP-labeled antibody	Visual detection	No
Tereshchenko et al., 2017	GVA-infected woody tissues	Unstable operation point	Immobilized GVA-antibodies	Optical/photoluminescence	No
Tereshchenko et al., 2020	GVA-infected woody tissues	Detection without dose-response curve	Silicon/ZnO-NRs/anti-GVA immune-sensing	Optical/Whispering Gallery Mode Resonators	No
Vashpanov et al., 2008	Purified ToRSV and GFLV	Detection without dose-response curve	Absorption on mesoporous silicon	Changes in drain-source current	No

**Table 3:** Test results (+ = positive/detectable, − = negative/undetectable, n.p. = not performed) from analysis carried out on GLRaV-3 and GFLV samples. A comparison of results reveals a higher sensitivity of LOC with respect to ELISA technique.

Sample Dilution	GLRaV-3		GFLV	
	ELISA	LOC	ELISA	LOC
1:3	+	n.p.	+	n.p.
1:5	+	n.p.	−	+
1:10	−	+	−	+
1:20	−	+	−	+
1:50	−	+	−	+
1:100	−	+	−	−

## Data Availability

Data is contained within the article.

## References

[1] Martelli G.P. (2014). Grapevine leafroll. J. Plant Pathol..

[2] Martelli G.P. (2014). Infectious degeneration (Grapevine fanleaf virus). J. Plant Pathol..

[3] Almeida R.P.P., Daane K.M., Bell V.A., Eblaisdell G.K., Cooper M.L., Eherrbach E., Epietersen G. (2013). Ecology and management of grapevine leafroll disease. Front. Microbiol..

[4] Maree H.J., Almeida R.P.P., Bester R., Chooi K.M., Cohen D., Dolja V.V., Fuchs M.F., Golino D.A., Jooste A.E.C., Martelli G.P. (2013). Grapevine leafroll-associated virus 3. Front. Microbiol..

[5] Freeborough M.-J., Burger J. (2006). Leaf Roll: Economic Implications. Wynboer. http://www.wynboer.co.za/recentarticles/200812-leafroll.php3.

[6] Nimmo-Bell The Economic Effects and Financial Impact of GLRaV3. http://www.nzwine.com/the-economic-effects-and-financialimpact/;jsessionid=D7464D6CE6F261FF32EEB266A042DB53.

[7] Martelli G.P., Savino V., Pearson R., Goheen A.C. (1990). Fanleaf degeneration. Compendium of Grape Diseases.

[8] Panno S., Caruso A., Bertacca S., Pisciotta A., Lorenzo R., Marchione S., Matić S., Davino S. (2021). Genetic Structure and Molecular Variability of Grapevine Fanleaf Virus in Sicily. Agriculture.

[9] Andret-Link P., Hoffmann C., Valat L., Ritzenthaler C., Demangeat G., Vigne E., Laval V., Pfeiffer P., Stussi-Garaud C., Fuchs M. (2004). Grapevine fanleaf virus: Still a major threat to the grapevine industry. J. Plant Pathol..

[10] Tsai C.-W., Rowhani A., Golino D.A., Daane K.M., Almeida R.P.P. (2010). Mealybug Transmission of Grapevine Leafroll Viruses: An Analysis of Virus-Vector Specificity. Phytopathology.

[11] Le Maguet J., Beuve M., Herrbach E., Lemaire O. (2012). Transmission of Six Ampeloviruses and Two Vitiviruses to Grapevine by Phenacoccus aceris. Phytopathology.

[12] (2020). Wine Production—Oiv First Estimates. http://www.oiv.int/public/medias/7541/en-oiv-2020-world-wine-production-first-estimates.pdf.

[13] EUR-Lex Council Directive 68/193/EEC of 9 April 1968 on the Marketing of Material for the Vegetative Propagation of the Vine. http://data.europa.eu/eli/dir/1968/193/oj.

[14] EUR-Lex Council Directive 2002/11/EC of 14 February 2002 Amending Directive 68/193/EEC on the Marketing of Material for the Vegetative Propagation of the Vine and Repealing Directive 74/649/EEC. http://data.europa.eu/eli/dir/2002/11/oj.

[15] EUR-Lex Commission Directive 2005/43/EC of 23 June 2005 Amending the Annexes to Council Directive 68/193/EEC on the Marketing of Material for the Vegetative Propagation of the Vine. http://data.europa.eu/eli/dir/2005/43/oj.

[16] Rowhani A., Uyemoto J.K., Golino D.A., Martelli G.P. (2005). Pathogen Testing and Certification of Vitis and Prunus Species. Annu. Rev. Phytopathol..

[17] Naidu R., Rowhani A., Fuchs M., Golino D., Martelli G.P. (2014). Grapevine Leafroll: A Complex Viral Disease Affecting a High-Value Fruit Crop. Plant Dis..

[18] Julich S., Riedel M., Kielpinski M., Urban M., Kretschmer R., Wagner S., Fritzsche W., Henkel T., Möller R., Werres S. (2011). Development of a lab-on-a-chip device for diagnosis of plant pathogens. Biosens. Bioelectron..

[19] Nezhad A.S. (2014). Future of portable devices for plant pathogen diagnosis. Lab Chip.

[20] Chiriacò M.S., Luvisi A., Primiceri E., Sabella E., De Bellis L., Maruccio G. (2018). Development of a lab-on-a-chip method for rapid assay of Xylella fastidiosa subsp. pauca strain CoDiRO. Sci. Rep..

[21] Rizzato S., Leo A., Monteduro A.G., Chiriacò M.S., Primiceri E., Sirsi F., Milone A., Maruccio G. (2020). Advances in the Development of Innovative Sensor Platforms for Field Analysis. Micromachines.

[22] Buja I., Sabella E., Monteduro A.G., Chiriacò M.S., De Bellis L., Luvisi A., Maruccio G. (2021). Advances in Plant Disease Detection and Monitoring: From Traditional Assays to In-Field Diagnostics. Sensors.

[23] Khater M., de la Escosura-Muñiz A., Quesada-González D., Merkoçi A. (2019). Electrochemical detection of plant virus using gold nanoparticle-modified electrodes. Anal. Chim. Acta.

[24] Jarocka U., Wąsowicz M., Radecka H., Malinowski T., Michalczuk L., Radecki J. (2011). Impedimetric Immunosensor for Detection of Plum Pox Virus in Plant Extracts. Electroanalysis.

[25] Jarocka U., Radecka H., Malinowski T., Michalczuk L., Radecki J. (2013). Detection of Prunus Necrotic Ringspot Virus in Plant Extracts with Impedimetric Immunosensor based on Glassy Carbon Electrode. Electroanalysis.

[26] Byzova N.A., Vinogradova S.V., Porotikova E.V., Terekhova U.D., Zherdev A.V., Dzantiev B.B. (2018). Lateral Flow Immunoassay for Rapid Detection of Grapevine Leafroll-Associated Virus. Biosensors.

[27] Tereshchenko A., Fedorenko V., Smyntyna V., Konup I., Konup A., Eriksson M., Yakimova R., Ramanavicius A., Balme S., Bechelany M. (2017). ZnO films formed by atomic layer deposition as an optical biosensor platform for the detection of Grapevine virus A-type proteins. Biosens. Bioelectron..

[28] Tereshchenko A., Yazdi G.R., Konup I., Smyntyna V., Khranovskyy V., Yakimova R., Ramanavicius A. (2020). Application of ZnO Nanorods Based Whispering Gallery Mode Resonator in Optical Immunosensors. Colloids Surf. B Biointerfaces.

[29] Vashpanov Y., Son J.Y., Kwack K.D. (2008). Mesoporous Silicon with Modified Surface for Plant Viruses and Their Protein Particle Sensing. Sensors.

[30] Fang X., Chen H., Yu S., Jiang X., Kong J. (2011). Predicting Viruses Accurately by a Multiplex Microfluidic Loop-Mediated Isothermal Amplification Chip. Anal. Chem..

[31] Piccinno E., Monteduro A.G., Dituri F., Rizzato S., Giannelli G., Maruccio G. (2021). Validation of a Lab-on-Chip Assay for Measuring Sorafenib Effectiveness on HCC Cell Proliferation. Int. J. Mol. Sci..

[32] Tourlousse D.M., Ahmad F., Stedtfeld R.D., Seyrig G., Tiedje J.M., Hashsham S.A. (2012). A polymer microfluidic chip for quantitative detection of multiple water- and foodborne pathogens using real-time fluorogenic loop-mediated isothermal amplification. Biomed. Microdevices.

[33] Chiriacò M.S., de Feo F., Primiceri E., Monteduro A.G., de Benedetto G.E., Pennetta A., Rinaldi R., Maruccio G. (2015). Portable gliadin-immunochip for contamination control on the food production chain. Talanta.

[34] Primiceri E., Chiriacò M.S., de Feo F., Santovito E., Fusco V., Maruccio G. (2016). A multipurpose biochip for food pathogen detection. Anal. Methods.

[35] Katz E., Willner I. (2003). Probing Biomolecular Interactions at Conductive and Semiconductive Surfaces by Impedance Spectroscopy: Routes to Impedimetric Immunosensors, DNA-Sensors, and Enzyme Biosensors. Electroanalysis.

[36] Chiriacò M.S., Primiceri E., Monteduro A.G., Bove A., Leporatti S., Capello M., Ferri-Borgogno S., Rinaldi R., Novelli F., Maruccio G. (2013). Towards pancreatic cancer diagnosis using EIS biochips. Lab Chip.

[37] Chiriacò M.S., Primiceri E., De Feo F., Montanaro A., Monteduro A.G., Tinelli A., Megha M., Carati D., Maruccio G. (2016). Simultaneous detection of multiple lower genital tract pathogens by an impedimetric immunochip. Biosens. Bioelectron..

[38] King A.M.Q., Adams M.J., Carstens E.B., Lefkowitz E.J. (2011). Virus Taxonomy. Ninth Report of the International Committee on Taxonomy of Viruses.

